# Association of intestinal and systemic inflammatory biomarkers with immune reconstitution in HIV+ patients on ART

**DOI:** 10.1186/s12950-020-00262-4

**Published:** 2020-10-15

**Authors:** Mariana del Rocio Ruiz-Briseño, Judith Carolina De Arcos-Jiménez, Sarah Ratkovich-González, Karina Sánchez-Reyes, Luz A. González-Hernández, Jaime F. Andrade-Villanueva, Monserrat Alvarez-Zavala

**Affiliations:** 1 Molecular Biology in Medicine PhD Program, Universidad de Guadalajara, Guadalajara, Jalisco Mexico; 2 HIV and Immunodeficiencies Research Institute (InIVIH), Universidad de Guadalajara, Guadalajara, Jalisco Mexico; 3 HIV Unit Department, Antiguo Hospital Civil de Guadalajara “Fray Antonio Alcalde”, Guadalajara, Jalisco Mexico

**Keywords:** HIV, Non-immune reconstitution, Biomarker, Immune activation, Inflammation, Gut damage, Proinflammatory cytokines

## Abstract

**Background:**

HIV infection is characterized by CD4^+^ T-cells depletion related to gut damage, microbial translocation, immune activation and intestinal and systemic low-grade inflammation. With the use of antiretroviral treatment, these alterations in HIV+ patients reach similar levels to HIV- controls. However, almost 20% patients have deficient immune reconstitution of CD4^+^ T-cells, which make them more susceptible to develop non-AIDS and AIDS comorbidities.

**Methods:**

HIV+ patients on ART, with sustained virologic control were grouped according to their immune reconstitution as: immunological responders (*n* = 18) and immunological non-responders (*n* = 18); also, HIV- controls were enrolled (*n* = 14). CD4^+^ and CD8^+^ T-cell activation (HLA-DR^+^ and CD38^+^ single and co-expression) were measured by flow cytometry. Serum levels of sCD14, sCD163, lipopolysaccharide, I-FABP, sST2, as well as fecal levels of calprotectin, lactoferrin and secretory IgA were evaluated by ELISA. Levels of C-reactive protein were determined by a high sensibility singleplex bead-based immunoassay. Serum and fecal concentrations of proinflammatory cytokines were quantified by multiplex bead-based immunoassay.

**Results:**

HLA-DR^+^ and CD38^+^ co-expression, as well as median fluorescence intensity in CD4^+^ and CD8^+^ T-cells subpopulations was greater in immunological non-responders group, after normalization and fold change calculation. Similarly, this group presented higher levels of sCD14, C-reactive protein, as well as fecal calprotectin and lactoferrin. Furthermore, both HIV+ groups showed elevated levels of proinflammatory cytokines in stool.

**Conclusions:**

Our data suggests that despite the virologic control, HIV+ patients under treatment with deficient immune reconstitution showed elevation of both innate and T-cells immune activation, as well as intestinal and systemic inflammation. However, some patients with CD4^+^ T-cells count above 350 cells/μL also presented these alterations. Future studies are necessary to evaluate the dynamics of multiple systemic and intestinal biomarkers in diverse types of HIV+ patients, as such as their clinical impact.

## Introduction

The Human Immunodeficiency Virus (HIV) infection continues to be a severe public health problems. Since Antiretroviral Therapy (ART) introduction, the life expectancy of HIV+ patients has been prolonged [1, 2]. The depletion of CD4^+^ T-cells due to HIV infection occurs principally in the gut, which causes a great damage to the intestinal epithelium, favoring microbial translocation; this alteration can as well trigger the activation of both innate and adaptive immune response in gut and bloodstream. Thus, this chronic immune activation induces intestinal and systemic low-grade inflammation resulting in an enteropathy [3, 4]. After ART initiation, immune activation, proinflammatory cytokines, biomarkers of gut damage, microbial translocation and generalized inflammation decrease, but remain higher than in uninfected controls [1, 2, 5–10]. These local and systemic alterations contribute to disease progression and increase the risk of non-AIDS related co-morbidities such as cardiovascular and liver diseases, metabolic disorders, cancer, fractures and fragility [1, 2, 11].

On the other hand, absolute CD4^+^ T-cells count is one of the most important features for monitoring treatment response in HIV patients [12]. The immune reconstitution differs among HIV+ patients; almost 20% of patients on treatment fail to increase the CD4^+^ T-cell count to more than 350 cells/μL despite the virological suppression, these patients are classified as Immunological Non-Responders (INRs) and, the HIV+ patients that have reached more than 350 cells/μL are Immunological Responders (IRs) [13–15]. Some risk factors are related to deficient CD4^+^ T-cells recovery such as advanced age, low CD4^+^ T-cells nadir count, low CD4^+^/CD8^+^ ratio, active coinfections, chronic inflammation and gut damage [1]. Moreover, the immune activation and inflammation are higher in INRs which make them more susceptible to progressing into AIDS than IRs [14].

Diverse biomarkers have been investigated in order to understand the pathogenesis of HIV infection in patients under ART, as well as, to identify patients that have a higher risk of deficient immune reconstitution and development of non-AIDS co-morbidities [16]. However, the pathogenesis of HIV infection is complex and multifactorial [16, 17].

Therefore, the aim of the study was to evaluate biomarkers of intestinal and systemic immune activation and inflammation, biomarkers of microbial translocation and gut damage, levels of cytokines related to inflammasome in gut and bloodstream, as well as, gut humoral activation, and their association with the immune reconstitution in HIV+ patients on ART.

## Methods

### Subjects

A cross-sectional study including HIV+ ART treated patients with sustained virologic control (viral load < 40 copies/mL) for more than 1 year were recruited from the HIV Unit Department, University Hospital “Fray Antonio Alcalde”, Guadalajara, Mexico from October 2017 to December 2018. They were grouped according to the threshold of 350 cells/μL of absolute CD4^+^ T-cells count in: Immunological Responders (*n* = 18) and Immunological Non-Responders (*n* = 18), as well as, HIV- controls (age matched) were enrolled (*n* = 14). Subjects who have reported use of antibiotics, probiotics or prebiotics within the previous month, individuals with a Body Mass Index (BMI) ≥30 and ≤ 19, also patients who presented opportunistic infections and co-infections with Hepatitis B and C virus were excluded. The investigation was approved by the Ethics Committee from University Hospital “Fray Antonio Alcalde” (approval number: 126/17). Signed informed consent was obtained from each subject prior to the recruitment. The CD4^+^ and CD8^+^ T-cells absolute count was determined with FACSCalibur System, BD; also, HIV-1 RNA viral load in plasma was measured through ROCHE Amplicor HIV-1 Monitor 1.5 Ultrasensitive PCR technique with COBAS Ampli-Prep/Cobas Taqman in the State Reference Laboratory.

### Sample collection

Plasma, serum and stool samples were obtained immediately after recruitment of each subject. Briefly, plasma sample was collected in EDTA tube and serum sample was obtained in serum separator tube where the sample was let to clot approximately 10 min at room temperature. Both sample tubes were centrifuged at 1600 *g* for 10 min. Next, plasma, serum and stool samples were aliquoted and stored at − 80 °C until use. For the quantification of intestinal proinflammatory cytokines, an aliquot of each stool sample was stabilized with phosphate buffer saline (PBS) and protease inhibitor cocktail (Promega, Madison, WI, USA), maintaining a 1:2 relation. The stabilized stool samples were centrifuged at 10,000 *g* for 15 min at 4 °C. Finally, the supernatant was collected and stored at − 80 °C until use.

### Systemic immune activation: HLA-DR^+^ and CD38^+^ co-expression in CD4^+^ and CD8^+^ T-cells, CD4^+^/CD8^+^ ratio, sCD14 and sCD163

T cell immunophenotyping and CD4^+^ and CD8^+^ T-cells relative count was performed on fresh blood samples anticoagulated with EDTA, using the antibodies PerCP anti-human CD3 (Clone: HIT3a), PE anti-human CD4 (Clone: RPA-T4), APC anti-human CD8 (Clone: RPA-T8); the samples were stained with Alexa 700 anti-human HLA-DR (Clone: L243) and FITC anti-human CD38 (Clone: HB-7) (All antibodies from BioLegend, San Diego CA, USA) to determine the immune activation. Attune NxT Flow Cytometer (Thermo Fisher Walthman, MA, USA) was used to acquire 10,000 events in the lymphocyte gate. The data was analyzed with Attune Nxt Flow Cytometer Software version 2.6 (Thermo Fisher, Walthman, MA, USA). The normalization was determined by the proportion of the HLA-DR^+^, CD38^+^ as well as HLA-DR^+^ and CD38^+^ expression or MFI with the CD4^+^ and CD8^+^ T-cells relative count, respectively. The fold change analysis was calculated by the log_2_ fold difference of HLA-DR^+^, CD38^+^ and, HLA-DR^+^ and CD38^+^ expression and MFI in CD4^+^ and CD8^+^ T-cells of HIV+ groups divided by the expression or MFI of the same cell subpopulations in HIV- controls [18, 19]. The CD4^+^/CD8^+^ ratio was calculated using the previously determined absolute CD4^+^ and CD8^+^ T-cells count. Serum levels of sCD14 and sCD163 were quantified by ELISA (both CUSABIO, Houston, TX, USA) according to the manufacturer instructions.

### Systemic inflammation and microbial translocation: high sensitive C-reactive protein and lipopolysaccharide

Singleplex bead-based immunoassay was used to measure serum levels of high sensitivity C-Reactive Protein (hsCRP) (LEGENDplex Human Vascular Inflammation Panel 1-CRP; BioLegend, San Diego, CA, USA) following the manufacturer protocol. Attune NxT Flow Cytometer (Thermo Fisher Walthman, MA, USA) was used to acquire 300 events. Analysis was performed in LEGENDplex Data Analysis Software v8 (BioLegend, San Diego, CA, USA). Furthermore, concentration of lipopolysaccharide (LPS) in serum was measured using Human Lipopolysaccharide ELISA kit (CUSABIO, Houston, TX, USA), according to manufacturer instructions.

### Proinflammatory cytokines quantification: IL-1β, IL-8 and IL-18

Multiplex bead-based immunoassay (LEGENDplex Human Inflammation Panel; BioLegend, San Diego, CA, USA) was used to quantify serum, as well as fecal cytokine levels (IL-1β, IL-8 and IL-18) employing the stabilized stool supernatant previously stored (see, Sample collection in Methods), following the manufacturer’s protocol. Attune NxT Flow Cytometer (Thermo Fisher Walthman, MA, USA) was used to acquire 300 events per each analyte. Data Analysis was performed in LEGENDplex Data Analysis Software v8 (BioLegend, San Diego, CA, USA).

### Gut damage and inflammation: I-FABP, sST2, sIgA, Calprotectin and Lactoferrin

Serum concentration of Intestinal Fatty Acids-Binding Protein (I-FABP) and the soluble IL-33 receptor sST2 were measured by ELISA (Human Intestinal Fatty Acid Binding Protein I-FABP ELISA kit; CUSABIO, Houston, TX, USA and Human ST2/IL-33R Quantikine ELISA kit, R&D Systems, Minneapolis, MN, USA), following each manufacturer protocols. Also, fecal levels of secretory IgA (sIgA), calprotectin and lactoferrin were determined by ELISA, employing the following kit: Secretory IgA ELISA, sIgA (ALPCO, Salem, NH, USA), Human Lactoferrin and Human Calprotectin (both Hycult Biotech, Uden, the Netherlands) according to manufacturer instructions.

### Statistical analysis

Proportions were compared using the Chi-square test. Group comparisons for biomarkers levels were performed using the Mann-Whitney U-test or Kruskal-Wallis test with Bonferroni-Dunn correction, depending of the groups number. Spearman’s correlations were done to determine the relationship between the concentration of the different evaluated biomarkers. For additionally analysis, levels of biomarkers above the 75th percentile were considered elevated. All statistics were calculated using IBM SPSS Statistics for Windows version 24 (IBM Corp., Armonk, NY, USA); plots were drawn with GraphPad Prism version 6 (GraphPad Software, La Jolla, CA, USA). Difference in biomarkers levels or correlations were considered statistically significant if *p <  0.05.*

## Results

### Clinical and demographic characteristics

The clinical and demographic characteristics of all participants are indicated in Table 1. All groups were paired by age but not by gender, having fewer males in the control group compared to INRs (*p <  0.05*). Healthy controls had a significant higher BMI versus INRs (*p <  0.05*); it is noteworthy that BMI from the control group fit into the category of overweight compared to all HIV+ patients which were in a normal weight rank. In relation to HIV+ patients, no statistical differences were observed in nadir CD4^+^ T-cell count and CD8^+^ T-cell count. Nevertheless, the INRs presented a CD4^+^/CD8^+^ ratio lesser than IRs (*p <  0.001*); furthermore, they had fewer years on ART (*p <  0.01*). Also, no difference were found in the type of ART used in both HIV+ groups, being ATRIPLA the most used.

**Table 1:** Clinical and demographic characteristics of participants

Variable	HIV- (***n = 14***)	IR (***n = 18***)	INR (***n = 18***)	***p*** value
Age, years, median (IQR)	33 (31.0–44.0)	39 (35.0–42.0)	44 (38.0–46.0)	0.089^a^
Male gender, *N* (%)	9 (64.3%)	13 (72.2%)	18 (100%)	*< 0.05*^*b*^
BMI, kg/m^2^; median (IQR)	26.1 (25.1–27.7)	23.6 (21.7–26.9)	22.5 (20.8–24.6)	*< 0.01*^*a*^
CD4^+^ T-cell count, cells/μL; median (IQR)	–	558.5 (492.0–705.0)	168 (141.0–256.0)	*< 0.001*^*c*^
Nadir CD4^+^ T-cell count, cells/μL; median (IQR)	-	69 (25–363.0)	42 (16.0–72.0)	0.055^c^
Nadir CD4^+^ T-cell count, cells/μL; mode	-	58	14	*< 0.01*^*b*^
CD8^+^ T-cell count, cells/μL; median (IQR)	–	703.5 (619.0–950.0)	683.5 (588.0–1000.0)	0.899^c^
CD4^+^/CD8^+^ ratio; median (IQR)	–	0.8 (0.6–1.1)	0.21 (0.2–0.4)	*< 0.001*^*c*^
Duration of HIV infection, years; median (IQR)	–	5.7 (3.3–10.0)	3.28 (1.1–6.9)	0.066^c^
Duration of ART, years; median (IQR)	–	5.5 (2.9–9.39)	2.8 (1.1–6.6)	*< 0.05*^*c*^

^a^Kruskall-Wallis test with Bonferroni correction, ^b^Chi square; ^c^Mann-Whitney U test

### Systemic immune activation

**Fig. 1:**
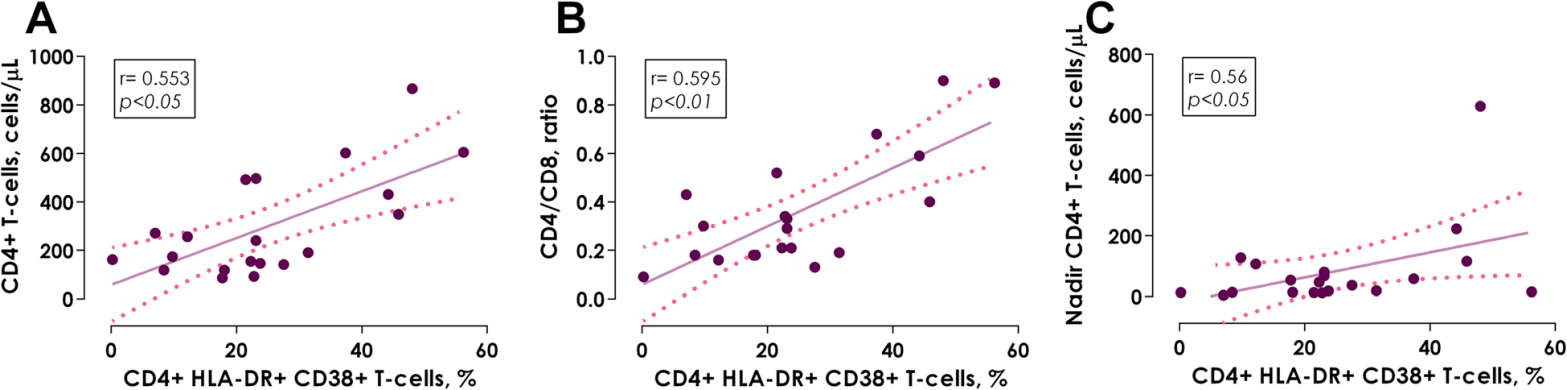
Association of HLA-DR^+^and CD38^+^ co-expression in CD4^+^ T-cells with the immune status of HIV+ ART treated patients. **a** HLA-DR^+^/and CD38^+^ co-expression in CD4^+^ T-cells versus absolute CD4^+^ T-cells count, **b** HLA-DR^+^ and CD38^+^ co-expression in CD4^+^ T-cells versus CD4^+^/CD8^+^ ratio and **c** HLA-DR^+^ and CD38^+^ co-expression in CD4^+^ T-cells versus nadir CD4^+^ T-cells count. Spearman correlation test

The T-cells immune activation was determined by HLA-DR^+^ and CD38^+^ single and co-expression, including Median Fluorescence Intensity (MFI) in CD4^+^ and CD8^+^ lymphocytes. As regards of the single expression of HLA-DR^+^, only the IRs had higher levels of MFI in CD8^+^ T-cells versus HIV- controls (*p <  0.05*). On the other hand, the INRs presented a lower percentage of CD38^+^ expression, as well as HLA-DR^+^ and CD38^+^ co-expression in CD4^+^ T-cells (*p <  0.05* and *p <  0.01*, respectively) in comparison with HIV- controls (Table 2). Positive correlations were found in HIV+ patients on ART between HLA-DR^+^ and CD38^+^ co-expression in CD4^+^ T-cells and absolute CD4^+^ T-cells count, CD4^+^/CD8^+^ ratio, as well as nadir CD4^+^ T-cell count (Fig. 1a, *r* = 0.553, *p <  0.05*; Fig. 1b, *r* = 0.595, *p <  0.01* and Fig. 1c, *r* = 0.56, *p <  0.05*, respectively); these associations suggest that, the reduction of the activating CD4^+^ T-cell profile is related to low absolute count of INRs (168 cells/μL) (Table 1).

**Table 2:** Systemic immune activation, inflammation, microbial translocation, proinflammatory cytokines, intestinal damage and inflammation biomarkers

	HIV- (***n = 14***)	IR (***n = 18***)	INR (***n = 18***)	***p*** value
**T-CELL IMMUNE ACTIVATION**				
***CD4***^***+***^ ***T-cells***				
HLA-DR^+^ expression, %	59 (16.2–92.9)	61.8 (22.7–84.3)	58.1 (37.3–75.8)	0.949^a^
HLA-DR^+^, MFI	7500 (6646–10,826)	9874 (5474–14,796)	7067 (5591–13,789)	0.802^a^
CD38^+^ expression, %	51.2 (42.9–56.1)	47.3 (35.2–54.6)	39.1 (25.3–42.7)	*< 0.05*^a^
CD38^+^, MFI	5203 (4322–6001)	4771 (4574–5666)	4930 (4475.5–5766.5)	0.892^a^
HLA-DR^+^ and CD38^+^ co-expression, %	34.7 (31.9–52.3)	40.8 (23.1–48.1)	18.1 (9.8–23.8)	*< 0.01*^a^
HLA-DR^+^ and CD38^+^, MFI	11,380 (7002.5–12,585.5)	5310 (4657–24,752)	9661 (7280–18,114)	0.727^a^
***CD8***^***+***^ ***T-cells***				
HLA-DR^+^ expression, %	37.8 (30.8–45.5)	23 (13.4–57.9)	35 (16.7–57.5)	0.648^a^
HLA-DR^+^, MFI	7019.5 (5789–8557)	10,965 (9207–17,730)	9696 (7193.5–12,288.5)	*< 0.05*^a^
CD38^+^ expression, %	46 (33.2–50.4)	33.6 (23–50.3)	36.2 (24–43.4)	0.618^a^
CD38^+^, MFI	4098 (3250–5171)	3724 (3342–3791)	3684 (3360–4640)	0.712^a^
HLA-DR^+^ and CD38^+^ co-expression, %	11.1 (6.6–13.2)	13.8 (11.6–15.8)	12.4 (6.4–26.3)	0.466^a^
HLA-DR^+^ and CD38^+^, MFI	8840 (7816–12,020)	13,987 (10852–19,953)	14,066 (11643–18,748)	0.186^a^
**INNATE IMMUNE ACTIVATION**				
sCD14, ng/mL	55.6 (12.2–199.6)	184.7 (109.4–329.9)	238.8 (196.2–378.9)	*< 0.01*^a^
sCD163, ng/mL	192.1 (158.3–254.9)	242.5 (141.8–339.8)	230.8 (94.5–385.9)	0.789^a^
**SYSTEMIC INFLAMMATION**				
C-reactive protein, mg/dL	0.27 (0.16–1.04)	1.24 (0.67–2.09)	1.63 (0.96–4.53)	*< 0.05*^a^
C- reactive protein (≥ 3.0 mg/dL), *N* (%)	1 (7.1%)	3 (16.7%)	6 (33.3%)	0.168^b^
**MICROBIAL TRANSLOCATION**				
Lipopolysaccharide, pg/mL	1098.7 (182.8–2876.2)	588.6 (257.1–1931)	979.2 (409.9–2281.8)	0.644^a^
**PROINFLAMMATORY CYTOKINES**				
Fecal IL-1β, pg/mL	10.4 (10.4–11.7)	47.1 (16.9–114.4)	44 (28.9–176.3)	*< 0.001*^a^
Fecal IL-8, pg/mL	8.21 (8.2–16.6)	116.8 (21.9–234.7)	88.7 (43.8–204.5)	*< 0.001*^a^
Fecal IL-18, pg/mL	40.7 (40.7–40.7)	66.8 (40.7–487)	132.8 (40.7454.6)	*< 0.01*^a^
IL-1β, pg/mL	11.1 (10.4–11.8)	11.8 (10.4–16.9)	16.9 (10.4–21.9)	0.368^a^
IL-8, pg/mL	16.58 (8.2–22)	16.6 (8.21–22)	16.6 (8.2–27.3)	0.845^a^
IL-18, pg/mL	89.7 (40.7–192.2)	158 (96.2–257.7)	173.4 (96.2–316.12)	0.153^a^
**INTESTINAL DAMAGE**				
I-FABP, ng/mL	6.33 (0.0–126.4)	31.2 (8.5–283.2)	53.6 (27.3–216.2)	0.164^a^
sST2, ng/mL	28.6 (23.6–35.6)	31.1 (23.4–39.3)	33.1 (27.6–38.5)	0.603^a^
**INTESTINAL INFLAMMATION**				
Fecal lactoferrin, μg/g	2.0 (1.8–2.5)	2.5 (1.9–2.9)	3.1 (2.2–4.0)	*< 0.05*^a^
Fecal lactoferrin (≥ 5.6 μg/g), *N* (%)	0 (0%)	1 (5.6%)	3 (17%)	0.202^b^
Fecal calprotectin, μg/g	2.7 (2.6–2.9)	2.7 (2.6–2.9)	3.1 (2.7–3.4)	*< 0.05*^a^
Secretory IgA, mg/L	8566.5 (5066.9–10,042.2)	10,122.1 (5721–10,844.8)	7791.2 (6257.4–11,329.9)	0.82^a^

^a^Kruskall-Wallis test with Bonferroni correction, ^b^Chi square. All values are expressed as median and interquartile range, except for those where another measurement expression is specified

**Fig. 2:**
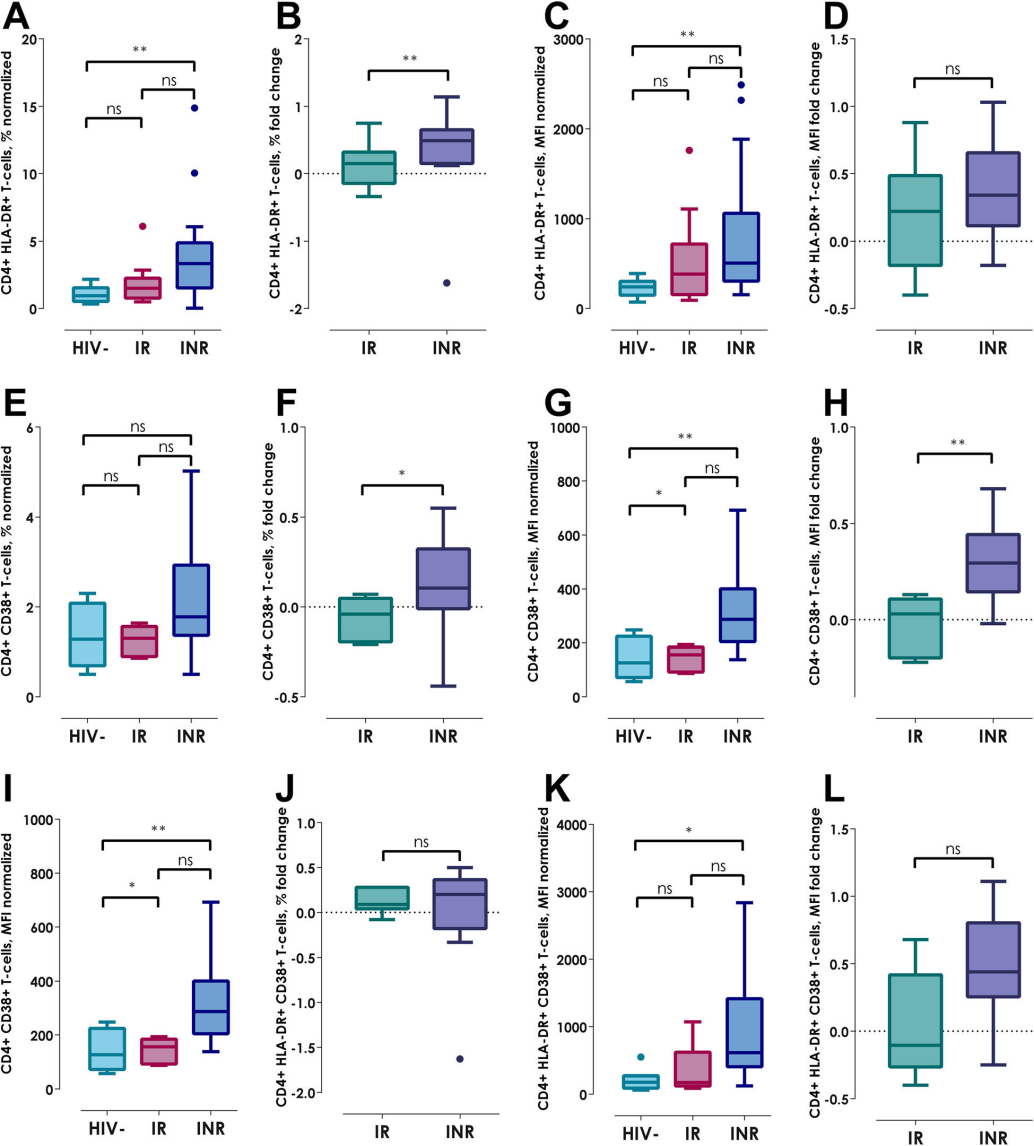
Normalization and fold change of expression of HLA-DR^+^ and CD38^+^ in CD4^+^ T-cells. **a** HLA-DR^+^ normalized expression, **b** HLA-DR^+^ fold change expression, **c** HLA-DR^+^ normalized MFI, **d** HLA-DR fold change MFI, **e** CD38^+^ normalized expression, **f** CD38^+^ fold change expression, **g** CD38^+^ normalized MFI, **h** CD38^+^ fold change MFI, **i** HLA-DR^+^ and CD38^+^ normalized co-expression, **j** HLA-DR^+^ and CD38^+^ fold change expression, **k** HLA-DR^+^ and CD38^+^ normalized MFI, **l** HLA-DR^+^ and CD38^+^ fold change MFI. Kruskall-Wallis test with Bonferroni correction for normalization graphs and Mann-Whitney U-test for fold change graphs. Data show as median (IQR); *p < 0.0 and **p < 0.01

Based on the previous data and since there was an important CD4^+^ and CD8^+^ T-cell count variation between the three groups, we performed a data normalization in order to analyze the single expression, HLA-DR^+^ and CD38^+^ co-expression and MFI, independently of the T-cells subpopulations count; in addition, based on this data we contrasted the fold change in both HIV+ groups. Regarding to CD4^+^ T-cells normalization, the INRs showed a higher HLA-DR^+^ expression and MFI (Fig. 2a and c, both *p <  0.01*), CD38^+^ MFI (Fig. 2g, *p < 0.01*) as well as HLA-DR^+^ and CD38^+^ MFI (Fig. 2k, *p < 0.05*); interestingly, by calculating the fold change, the INRs showed the same pattern in relation to HLA-DR^+^ expression (Fig. 2b, *p < 0.01*) and CD38^+^ in both expression and MFI in CD4^+^ T-cells (Fig. 2f, *p < 0.05* and 2H, *p <  0.01*) compared to IRs.

**Fig. 3:**
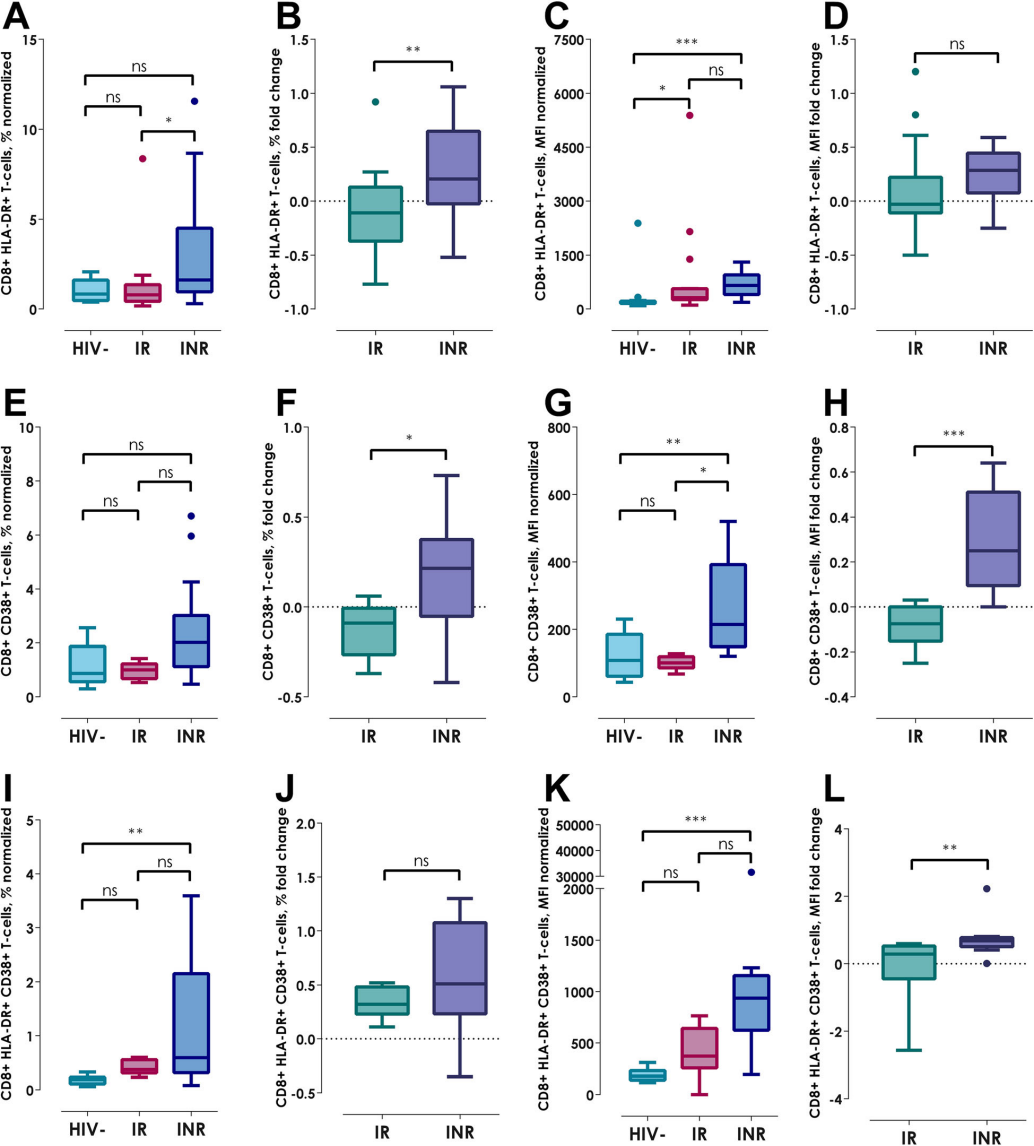
Normalization and fold change of expression of HLA-DR^+^ and CD38^+^ in CD8^+^ T-cells. **a** HLA-DR^+^ normalized expression, **b** HLA-DR^+^ fold change expression, **c** HLA-DR^+^ normalized MFI, **d** HLA-DR fold change MFI, **e** CD38^+^ normalized expression, **f** CD38^+^ fold change expression, **g** CD38^+^ normalized MFI, **h** CD38^+^ fold change MFI, **i** HLA-DR^+^ and CD38^+^ normalized co-expression, **j** HLA-DR^+^ and CD38^+^ fold change expression, **a** HLA-DR^+^ and CD38^+^ normalized MFI, B) HLA-DR^+^ and CD38^+^ fold change MFI. Kruskall-Wallis test with Bonferroni correction for normalization graphs and Mann-Whitney U-test for fold change graphs. Data show as median (IQR); *p < 0.05, **p < 0.01 and ***p < 0.001

Similarly, in CD8^+^ T-cells normalization, the INRs had higher HLA-DR^+^ expression and MFI (Fig. 3a, *p < 0.05*, 3C, *p <  0.001*), CD38^+^ MFI (Fig. 3g, *p < 0.01*), HLA-DR^+^ and CD38^+^ co-expression as well as MFI (Fig. 3i, *p < 0.01* and 3 K, *p <  0.001*, respectively); whereas, the IRs showed similar elevation to INRs in HLA-DR^+^ MFI of CD8^+^ T-cells (Fig. 3c, *p < 0.05*). Furthermore, comparing the fold change in CD8^+^ T-cells, the INRs presented an increase of HLA-DR^+^ expression (Fig. 3b, *p < 0.01*), CD38^+^ MFI (Fig. 3h, *p < 0.001*), also HLA-DR^+^ and CD38^+^ MFI (Fig. 3l, *p < 0.01*).

**Fig. 4:**
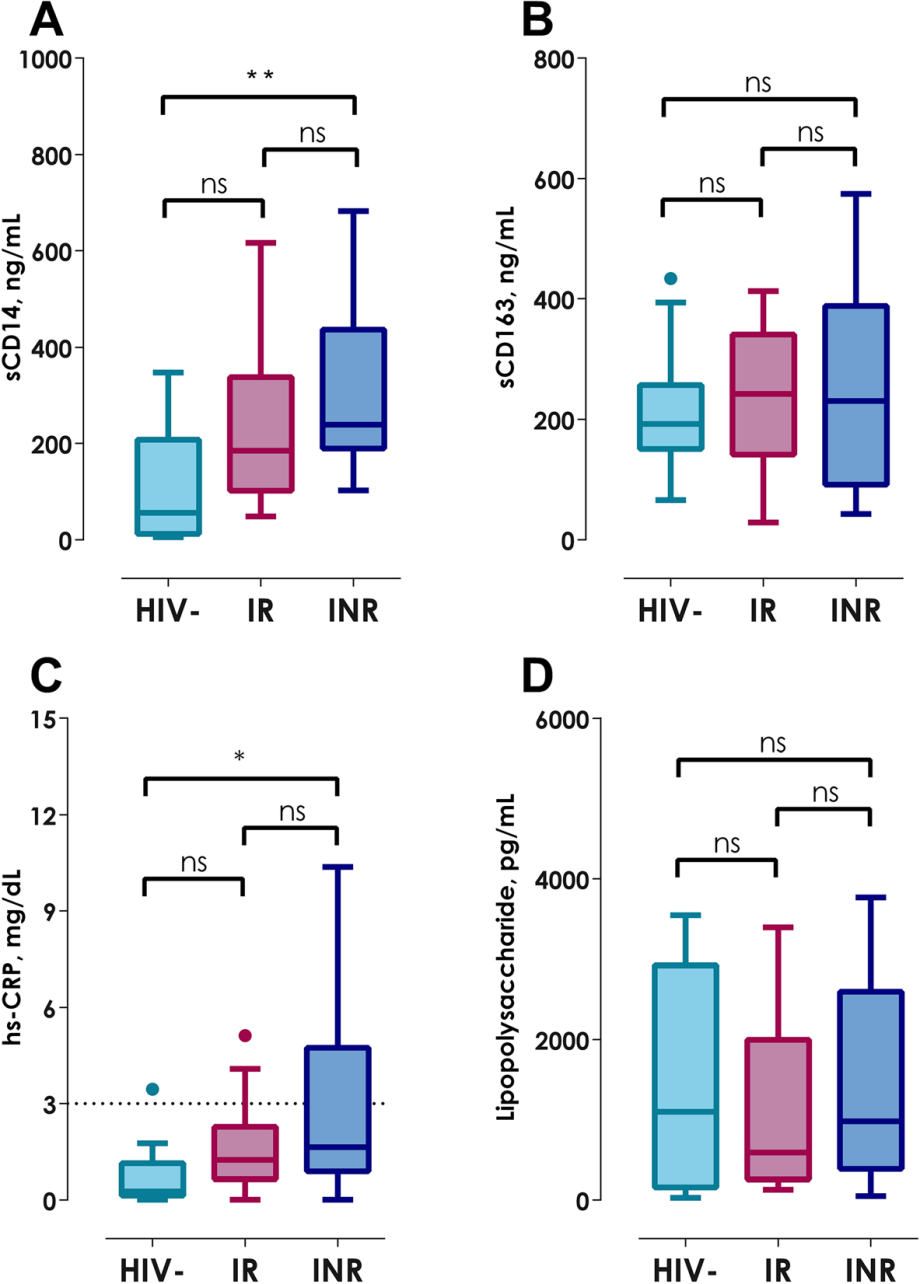
Quantification of innate immune activation, systemic inflammation and microbial translocation. **a** sCD14, **b** sCD163, **c** High sensitivity C-Reactive Protein and **d** Lipopolysaccharide. Kruskall-Wallis test with Bonferroni correction. Data show as median (IQR);ns: non-significant, *p < 0.05 and **p < 0.01

Regarding the innate immune activation, INRs had significantly higher sCD14 level in contrast to the HIV- group (Table 2; Fig. 4a, *p < 0.01*); also, 28% of IRs and 33% of INRs showed concentrations of sCD14 above 75th percentile. Nevertheless, all participants presented similar sCD163 amounts (Table 2; Fig. 4b, *p* = 0.789), however 28% of both HIV+ groups showed elevated levels of this biomarker.

### Systemic inflammation and microbial translocation

hsCRP is an inflammation biomarker widely used in several pathologies, including HIV infection; also, this acute reactant is related to disease progression and cardiovascular disease risk. In our study, the INRs showed significantly higher hsCRP levels in comparison with HIV- controls (Fig. 4c, *p < 0.05*), like sCD14 elevation previously observed. Importantly, hsCRP levels in 33% of INRs and 17% of IRs exceeded 3.0 mg/dL, cut-off value for higher risk of mortality and development of cardiovascular disease (Table 2) [20]. Despite the hsCRP is non-specific, their determination along with others biomarkers related to immune activation and inflammation, provide information about the systemic inflammatory state; importantly, it is known that the hsCRP is a surrogate marker of IL-6 [21]. In INR, the increase of this reactant of acute phase and the sCD14 suggest that, independently of the virologic control the systemic low-grade inflammation continues, which could be affect their capacity of recovery CD4^+^ T-cells. In addition, LPS was evaluated as microbial translocation biomarker. All study groups presented high variability in LPS levels, including HIV- controls; thus, no statistical significances were found (Table 2; Fig. 4d, *p* = 0.644).

### Proinflammatory cytokines

**Fig. 5:**
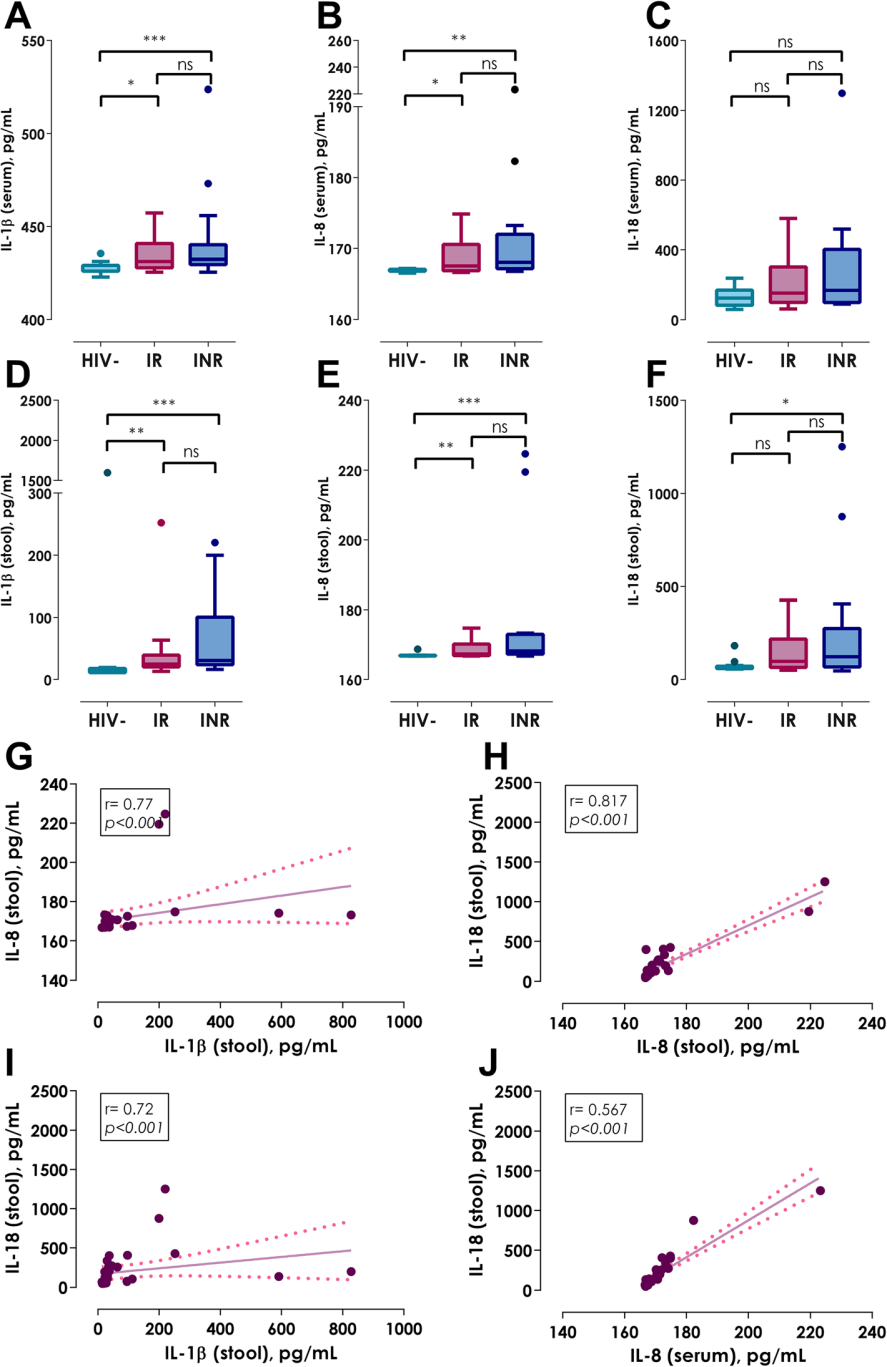
Quantification of systemic and intestinal proinflammatory cytokines. **a** Serum IL-1β, **b** Serum IL-8, **c** Serum IL-18, **d** Fecal IL-1β, **e** Fecal IL-8, **f** Fecal IL-18, **g** Association between fecal levels of IL-1β and IL-8, **h** Association between fecal levels of IL-8 and IL-18, **i** Association between fecal levels of IL-1β and IL-18 and **j** Association between serum levels of IL-8 and fecal IL-18. Kruskall-Wallis test with Bonferroni correction. Data show as median (IQR); *p < 0.05, **p < 0.01 and ***p < 0.001. Spearman correlation test

Considering the previous, the intestinal and systemic immune activation is an essential component of the immunopathogenesis of HIV infection, it was of our interest to evaluate the levels of proinflammatory cytokines related to the inflammasome (IL-1β, IL-8 and IL-18) in serum and stool samples. In bloodstream, all proinflammatory cytokines showed similar levels in all three groups (Table 2; Fig. 5a, *p* = 0.368; 5B *p* = 0.845 and 5C, *p* = 0.153). In contrast, HIV+ patients, independently of their immune reconstitution, had higher levels of IL-1β, IL-8 and IL-18 in stool supernatant in comparison with HIV- controls (Table 2; Fig. 5d, *p < 0.001*; 5E, *p <  0.001* and 5F, *p <  0.01*). Furthermore, the levels of IL-1β, IL-8 and IL-18 in stool showed correlation between them (Fig. 5g, IL-1β versus IL-8: *r* = 0.77, *p <  0.001*; Fig. 5h, IL-1β versus IL-18: *r* = 0.72, *p <  0.001*; Fig. 5i IL-8 versus IL-18: *r* = 0.817, *p <  0.001*), indicating a possible feedback among them, which could increase the gut inflammation. In addition, IL-18 fecal levels were associated with IL-8 and IL-18 serum levels (Fig. 5j, *r* = 0.567, *p < 0.001*; *r* = 0.449, *p < 0.01*); these data suggest that, higher levels of the intestinal proinflammatory cytokines indirectly promote systemic low-grade inflammation.

### Gut damage and inflammation

**Fig. 6:**
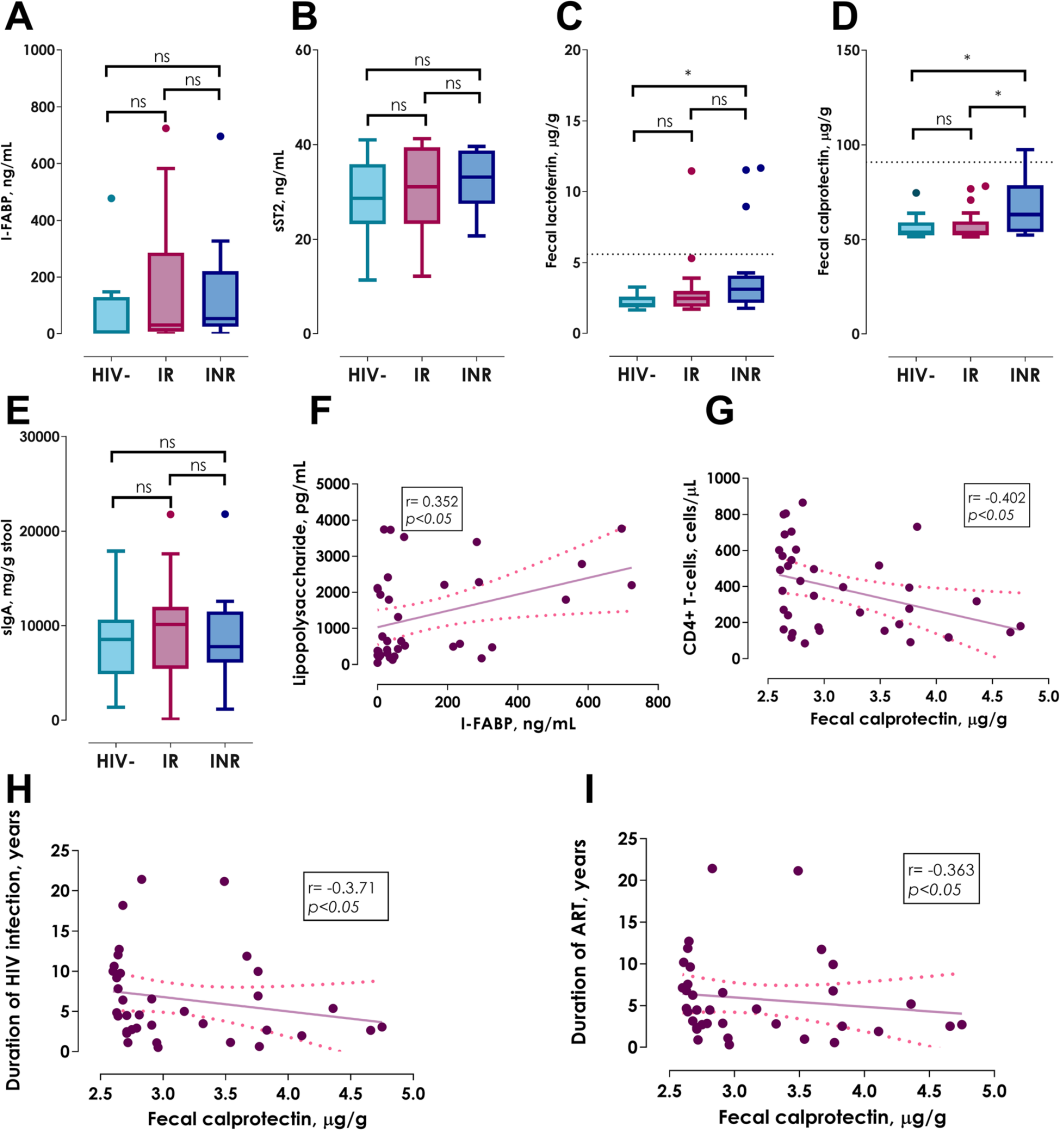
Determination of gut damage and inflammation. **a** I-FABP, **b** sST2, **c** Fecal lactoferrin, **d** Fecal calprotectin, **e** sIgA, **f** Association between I-FABP levels and Lipopolysaccharide concentration, **g** Association between fecal calprotectin levels and absolute CD4^+^ T-cells count, **h** Association between fecal calprotectin levels and duration of HIV infection and **i** Association between fecal calprotectin levels and duration of antiretroviral treatment. Kruskall-Wallis test with Bonferroni correction. Data show as median (IQR); *p < 0.05. Spearman correlation test

Since all HIV+ patients showed higher levels of proinflammatory cytokines in the gut, we evaluate intestinal mucosal damage in HIV+ patient on ART through the quantification of I-FABP and sST2 in serum. Surprisingly, no statistical difference was found in I-FABP concentration (Table 2; Fig. 6a, *p* = 0.164); however, 33% of INRs and 28% of IR presented levels of I-FABP above 75th percentile. Interestingly, a positive association between I-FABP and LPS levels (Fig. 6e, *r* = 0.352, *p < 0.05*) was observed, denoting the relationship between gut damage and microbial translocation in HIV infection. In the same way to I-FABP, all participants had similar concentration of sST2 (Table 2; Fig. 6b, *p* = 0.603); also, 28% of both HIV+ groups showed elevated levels of sST2.

In addition to the previous biomarkers, concentration of fecal lactoferrin and calprotectin were measured for a more detailed analysis of the intestinal environment. Particularly, INRs showed significantly elevated lactoferrin levels in comparison to HIV- controls (Table 2; Fig. 6c, *p < 0.05*). Regarding calprotectin levels, INRs showed a significant increase of this protein in comparison with HIV- controls and IRs (Table 2; Fig. 6d, *p < 0.05*, both comparisons). Despite the elevated levels of lactoferrin and calprotectin, none of HIV+ groups surpassed the established cut-off to determine intestinal inflammation in Inflammatory Bowel Disease (IBD) in non-HIV+ subjects: 7.25 μg/g of stool for lactoferrin and 90.85 μg/g for calprotectin (Table 2); however, 50 and 44% of INRs present elevated levels (above 75th percentile) of lactoferrin and calprotectin, respectively [22–24]. Moreover, calprotectin levels were negatively correlated with the absolute CD4^+^ T-cells count (Fig. 6g, *r* = − 0.402; *p < 0.05*), as well as with HIV infection and ART duration (Fig. 6h, *r* = − 0.371, *p < 0.05* and Fig. 6i, *r* = − 0.363, *p < 0.05*, respectively); which emphasises the impact of gut inflammation in the immune reconstitution of HIV+ patients despite ART. Finally, we evaluated the amount of sIgA in stool without finding a statistical difference between the three groups (Table 2; Fig. 6e, *p* = 0.82).

## Discussion

Due to the great advances in ART regarding HIV infection, the life prognostic of the people living with HIV have increased, however major challenges have overcome. Despite of the virological suppression achieved by ART, almost 20% of HIV patients fail to recover CD4^+^ T-cells counts above 350 cells/μL, these are called INRs [13]. As is widely known, the main risk for the development of non-AIDS comorbidities and AIDS progression is a low CD4^+^ T-cell count, along with the fact that ART is unable to control: the residual HIV replication in anatomical sanctuaries, immune activation and the onset of a subclinical chronic inflammatory state [15, 25, 26]. It has become a great necessity, to find a set of biomarkers in HIV+ patients on ART that can reflect the immune activation and low-grade chronic inflammation. Thus, this study was focused in measuring diverse biomarkers related to immune activation, systemic and gut inflammation, microbial translocation and gut damage, in order to determine their relationship with the immune reconstitution in HIV+ patients on ART. Particularly, we analyzed the inflammatory gut microenvironment by measuring proinflammatory cytokines levels in stool supernatant, through a noninvasive method and these data were compared with the systemic proinflammatory cytokines concentrations. Few studies have quantified the levels of cytokines in stool of HIV+ patients on ART. The methodology used in our study could be a good alternative as a noninvasive test, to measure the levels of intestinal proinflamatory cytokines in HIV+ patients. Particularly in those that, despite of achieving a virologic suppression showed a poor immune reconstitution which could develop non-AIDS comorbidities.

Since one of the major side effects in HIV infection is the Gut Associated Lymphoid Tissue (GALT) exhaustion; the unsuccessful recovery of this immunological niche has been related to an increased risk of microbial translocation and a trigger of the immune activation and low-grade chronic inflammation, even in the presence of ART [27].

Several other reports have shown a constant cellular immune activation in HIV patients on ART, this activation persists even after the immune recovery and achievement of virologic suppression. Over the past years different cellular biomarkers have been proposed to study the activation profile of the CD4^+^ and CD8^+^ T-cells. Currently, the nadir CD4^+^ T-cell count, the CD4^+^/CD8^+^ ratio, HLA-DR^+^ and CD38^+^ co-expression in CD8^+^ T-cells remain as the most relevant immunologic biomarkers for clinicians [25]. Regarding these biomarkers, we found that, though no differences in nadir CD4^+^ T-cells count between IRs and INRs, the INRs had a worst CD4^+^/CD8^+^ ratio (0.21). Furthermore, we characterized the remaining immune activation of the CD4^+^ and CD8^+^ T-cells, in IRs CD8^+^ T-cells remain highly activated (increased CD38^+^ MFI in CD8^+^ T-cells) despite the immune reconstitution and the aviremic state. However, due to the variability on CD4^+^ and CD8^+^ T-cell count, we were not able to detect a significant increase of T-cell activation in the INRs, therefore it was necessary to perform a normalized and fold change analysis. Through this analysis, it could be demonstrated that the co-expression of CD38^+^and HLA-DR^+^ in CD4^+^ and CD8^+^ T-cells, was higher in INRs, since both markers have been related to exhausted and activated T-cells [28]. We consider that this kind of analysis could be a good strategy to avoid the understimation of T-cells immune activation.

INRs have a highly activated and exhausted CD4^+^ and CD8^+^ T-cell profile, which increases their risk of an AIDS related comorbidity. Several studies have confirmed the remaining cellular activation in CD4^+^ and CD8^+^ T-cells in HIV+ patients on ART [10, 29, 30]; this feature still represents a challenge, since ART is not able to control the remaining immune activation. In elite controllers it has been proven that HIV infection increased the T-cell activation and the exhaustion of the immune system and, in the patients the use of ART improves these characteristics [31].

Our data showed that immune inflammation in HIV+ patients on ART is also reflected in innate immune activation. sCD14 has been considered as a biomarker of acute phase response, microbial translocation and monocyte/macrophage activation. This soluble marker was significantly higher in the INR group; particularly, 33% of these patients had levels above the 75th percentile. This elevation agrees with the T-cells immune activation, detected in this same group. Also, sCD14 is related to the immunopathogenesis of the HIV infection, higher plasmatic levels have been related to rapid progression of the disease. Currently, this biomarker above 75th percentile is an independent predictor of mortality mainly associated with neurocognitive impairment, cardiovascular disease and platelet activation, considering it as part of the prothrombogenic phenotype of the HIV+ patients [3, 32–34]. Other studies have tried to relate sCD14 levels with insulin resistance and diabetes onset, as well as other metabolic diseases in HIV patients; though significant high levels of sCD14 have been reported in HIV+ patients, none of these studies found an association with the disease or with hsCRP levels [3, 32]. Another biomarker of the innate immunity is sCD163, this soluble molecule is related to monocyte activation and inflammation, and is key in the atherosclerotic plaque formation. In the Multicenter AIDS Cohort Study (MACS) and other recent studies, it has been reported that levels of inflammation and immune activation biomarkers, including sCD163, decrease after ART initiation [33]. In our study, we have not detected significant differences in the concentrations of sCD163 among groups; however, this is consistent with previous reports that did not find persistently elevated levels of sCD163 in HIV+ patients on ART [32–36].

Considering the higher T-cells and innate immune activation and the increased levels of hsCRP, both HIV+ groups (IRs and INRs) present an inflammatory phenotype, despite the virologic control and independently, of CD4^+^ T-cells reconstitution. Interestingly, 17% of the IRs subjects and 33% of INRs patients exhibit levels above 3 mg/dL of hsCRP; this cut-off point is associated with higher risk of mortality and the development of cardiovascular diseases and opportunistic infections [20]. Particularly, the higher immune activation and systemic inflammation biomarkers levels (sST2 and hsCRP) and the low absolute CD4^+^ T-cell count could promote an increase of the risk of arterial hypertension and diastolic dysfunction in INRs [37].

Since most of the inflammatory biomarkers where elevated in both HIV+ groups when compared to controls, we suppose that systemic cytokine levels were increased in these patients; however, we did not find differences in serum proinflammatory cytokines. Several reports have shown that the use of ART helps to decrease the systemic proinflammatory cytokine levels in a time depending manner [38].

Our data showed a systemic inflammatory environment in both HIV+ groups, being higher in INR. Importantly, BMI values between 25 and 30 have been related with a better CD4^+^ T-cells recovery in HIV+ patients after ART initiation [39]. Despite that our INR group showed lower levels of BMI compared to IR and control group (which fit into the category of overweight), they presented higher levels of inflammation. This inflammation could be multifactorial and may not be directly attributed to the BMI. Considering the previous, we propose that this inflammation was probably stimulated by gut damage, microbial translocation, and an activation of immune system in gut. Thus, we measured indirect and direct biomarkers to evaluate the gut damage and inflammation, as well as microbial translocation. Since LPS is the main ligand of CD14, levels of LPS and sCD14 are often related to gut damage, microbial translocation and inflammation [3]. Contrary to the expected, we did not find a significant difference in LPS; nevertheless, other Toll-like receptors (TLR) ligands as well as proinflammatory cytokines can stimulate the release of sCD14 and its elevation does not necessarily reflect the immune activation by LPS since this molecule just represent Gram negative bacteria [40]. Regarding gut permeability, we measure I-FABP serum levels; even though no significant differences were detected, 28% of IRs and 33% of INRs showed elevated I-FABP levels, this might reflect established gut damage that exacerbates the inflammatory phenotype shown by this HIV+ group. This data differs to other previous reports that demonstrate increase I-FABP levels even in the presence of ART (it could be due to the small sample size); however, some of these studies relate the higher levels of I-FABP in HIV+ patients on ART to sugar and fatty acid intake, also half of this patients were under a protease inhibitor ART scheme [36, 38, 41].

In order to analyze the intestinal immune activation and inflammation, also fecal proinflammatory cytokines were measured. All HIV+ patients on ART showed a significant higher levels of IL-1β, IL-8 and IL-18, regardless of their capacity for immune reconstitution. This elevation of inflammasome dependent cytokines could be related to HIV residual replication and dysbiosis in the gut. Based on these results, we considered that the excessive gut inflammation impacts directly over dysfunctional adaptive and innate immune activation, as well as the systemic inflammatory state; whether this profile is sufficient to cause a bystander effect and contributes to the deficient immune reconstitution in the INRs remains unknown, and is clearly a mechanism that deserves to be elucidated. To our knowledge, this is the first study that investigate gut inflammation through cytokine levels with a noninvasive approach in HIV+ patients. Other groups have already reported high levels of IL-18, IL-1β, and IL-8 in gut biopsies from HIV+ patients; in this study, it was proposed that the IL-18 production is induced on intestinal epithelial cells by HIV which promotes intestinal permeability and microbial translocation [42]. Even though our result are similar to this research, this highlights the importance of measuring this biomarker at different anatomical compartments [7]. On the other hand, fecal cytokine levels were measured with a similar methodology in patients with Parkinson disease, finding a matching cytokine profile linked to gut inflammation [43].

Since IL-8 and IL-18 are considered as cytokines linked to production, migration, and diverse neutrophils effector functions, it is probable that these innate cells could be implicated in the characteristic gut damage and inflammation of HIV infection. Thus, we measure fecal calprotectin and lactoferrin, two of the main enzymes liberated by the neutrophils. Both molecules are noninvasive gut inflammatory biomarkers and they have been related to diverse gastrointestinal inflammatory diseases. Particularly in our study, we found that calprotectin and lactoferrin are elevated in both HIV+ groups and, 17% of the INR and 5.6% of IR exceed the cut-off point of lactoferrin associated to gut inflammation (5.6 μg/g of stool); although, calprotectin concentrations were below the levels that are considered pathological in IBD [22–24]. Unfortunately, IBD gut damage and the HIV enteropathy are evidently different (HIV infection is characterized by a depletion of Th17 cells, affecting neutrophil recruitment, mucosal regeneration and antimicrobial peptides production), thus we cannot consider that the levels found in our study are not pathological; importantly, 50% of INRs present elevated concentrations (75th percentile) of lactoferrin and calprotectin. To our knowledge, fecal calprotectin has been poorly studied in HIV+ subjects, a previous research by Pastor, L. et al. showed that IBD biomarkers (calprotectin, lactoferrin, sIgA, Zonulin-1) could not be used to measure gut inflammation in HIV patients; however, in this study other biomarkers such as proinflammatory cytokines levels, sCD14 levels, LPS, I-FABP were not measured [3]. Another investigation from Uganda, measured fecal calprotectin levels in HIV+ ART naïve children; these patients showed a high calprotectin concentration (above the reference value) and they correlated with disease progression [44]. Is worthy to mention that, in this study the low CD4^+^ T-cells count correlates with fecal calprotectin levels, just as in our INRs.

## Conclusions

In conclusion, HIV+ patients on ART presented higher levels of proinflammatory cytokines, which promotes the gut inflammation, despite having achieved virological suppression. The alteration of gut homeostasis can be reflected in the systemic T-cells and innate immune activation, which favors the chronic inflammation. The deficient immune reconstitution can be related to this persistent immune activation (high single and co-expression of HLA-DR^+^ and CD38^+^ in CD4^+^ and CD8^+^ T-cells) and inflammation state, particularly in the intestinal environment (high levels of fecal calprotectin and lactoferrin, as well as higher levels of sCD14, C-reactive protein); however, some HIV+ patients with CD4^+^ T-cells counts above 350 cells/μL also showed the same profile. Our data highlights the importance of biomarkers research in HIV infection, especially in the gut where damage is severe and partially reverted with the use of ART. Future studies should evaluate the dynamics of multiple biomarkers during HIV infection on diverse types of HIV+ patients considering a larger sample size than the one used in our study; in order to find the best combination of noninvasive biomarkers that can be used by the clinicians to evaluate and monitor the systemic and gut environment in HIV+ patients on ART.

## Data Availability

The datasets used and/or analysed during the current study are available from the corresponding author on reasonable request.

## Abbreviations

μg: micrograms
μL: microliters
AIDS: Acquired Immunodeficiency Syndrome
APC: Allophycocyanin
ART: antiretroviral treatment
BMI: Body Mass Index
CD: Cluster of Differentiation
hsCRP: high sensitivity C-Reactive Protein
dL: deciliters
EDTA: Ethylenediaminetetraacetic acid
FITC: Fluorescein Isothiocyanate
g: grams
*g*: gravities
GALT: Gut Associated Lymphoid Tissue
HIV: Human Immunodeficiency Virus
HLA-DR: Human Leukocyte Antigen-DR isotype
IBD: Inflammatory Bowel Disease
I-FABP: Intestinal Fatty Acid-Binding Protein
IL: Interleukin
INRs: Immunological Non-Responders
IQR: Interquartile range
IRs: Immunological Responders
kg: kilograms
LPS: Lipopolysaccharide
m: meters
MACS: Multicenter AIDS Cohort Study
MFI: Median Fluorescence Intensity
mg: milligrams
mL: milliliters
ng: nanograms
PBS: Phosphate Buffer Saline
PE: Phycoerythrin
PerCP: Peridinin-Chlorophyll-protein
pg: picograms
RNA: Ribonucleic acid
sIgA: secretory Immunoglobulin A
sST2: soluble ST2
TLR: Toll-like receptor
